# Emergency residents' self-perceived readiness for practice: the association of milestones, entrustable professional activities, and professional identities—a multi-institutional survey

**DOI:** 10.3389/fmed.2023.1032516

**Published:** 2023-05-12

**Authors:** Yu-Che Chang, Madalitso Khwepeya, Nothando S. Nkambule, Renee S. Chuang, Chung-Hsien Chaou

**Affiliations:** ^1^Chang Gung Medical Education Research Centre, Chang Gung Memorial Hospital, Taoyuan, Taiwan; ^2^Department of Emergency Medicine, Chang Gung Memorial Hospital, Taoyuan, Taiwan; ^3^College of Medicine, Chang Gung University, Taoyuan, Taiwan; ^4^International Graduate Program of Education and Human Development (IGPEHD), National Sun Yat-Sen University, Kaohsiung, Taiwan; ^5^Health Policy and Leadership Program, School of Public Health, Loma Linda University, Loma Linda, CA, United States

**Keywords:** competency-based medical education, competency-based learning, emergency medicine residents, entrustable professional activities, milestone, professional identity, residents, Taiwan

## Abstract

**Background:**

As a successful innovation, competency-based medical education and its assessment tools continue to be a key strategy in training future doctors and tracking their performance trajectories. Linked to professional identity, evidence suggests that clinical competence is related to thinking, acting and feeling like a physician. Thus, incorporating the values and attitudes of healthcare professions as part of their professional identity in the clinical workplace improves professional performance.

**Methods:**

Through a cross-sectional study, we examined the association of milestone, entrustable professional activities (EPA) and professional identity using self-reported tools among emergency medicine residents from 12 teaching hospitals across Taiwan. Milestone, EPA and professional identity were assessed using the Emergency Medicine Milestone Scale, Entrustable Professional Activity Scale and Emergency Physician Professional Identity and Value Scale, respectively.

**Results:**

The results of a Pearson correlation indicated a significant positive correlation between milestone-based core competencies and EPAs (*r* = 0.40 ~ 0.74, *p* < 0.01). The professional identity domain of skills acquisition, capabilities and practical wisdom was positively correlated with milestone-based core competencies of patient care, medical knowledge, practice-based learning and improvement, and system-based practice (*r* = 0.18 ~ 0.21, *p* ≤ 0.05), and six items of EPA (*r* = 0.16 ~ 0.22, *p* < 0.05). Additionally, the professional identity domain of professional recognition and self-esteem was positively correlated with practice-based learning and improvement, and system-based practice milestone competencies (*r* = 0.16 ~ 0.19, *p* < 0.05).

**Conclusion:**

This study demonstrates milestone and EPA assessment tools are highly linked and therefore, can be synergistically used by supervisors and clinical educators to evaluate clinical performance during residency training. Emergency physicians’ professional identity is partly influenced by the advancement of skills and a resident’s ability to learn, effectively perform tasks and make appropriate medical decisions at the system level in their clinical practice. Further research is warranted to understand the importance of residents’ competency in relation to their professional identity development trajectory during clinical training.

## Introduction

The implementation of competency-based medical education (CBME) to help medical trainees to smoothly transition into the clinical workplace and as a scaffold for readiness to practice is valuable (1, 2). Nonetheless, nowadays, it is anticipated for medical training programs to concurrently promote clinical competence and professional identity formation in clinical practice (3). This is because thinking, acting and feeling like a physician is key to professional development (4). Despite this proposed significance, the relationship between competence (doing) and professional identity (being or feeling like a physician) remains ambiguous. In this study, we argue that central to learning in preparation for readiness to practice, is the gradual participation of junior medical trainees within their community of practice through peripheral legitimate patient care under supervision to independent decision making in medical practice which enhances their sense of membership and professionalism (4, 5). This is consistent with Cruess et al. (4) conceptualization of the non-linear and complicated relationship between “doing” and “being” stages of practice. The conceptualization depicts how social interactions over time facilitate both the accumulating experiences and the internalization of professional attitudes, values and beliefs, necessary to help medical trainees transition into clinical practice. Consequently, the construction of professional identity represents the final stage in the trajectory of professional development; helping medical trainees to build strong relationships with their co-workers, belongingness in their community of practice which promote confidence in themselves, and a better sense of independence (5–7). Drawing from Cruess et al. (4) amended framework of Miller’s pyramid, this study aims to investigate the association between “doing” and “being” a healthcare professional.

By examining this relationship, our study serves as a response to Cruess’s call to assess the association between competencies and professional identities among healthcare professionals. Both competencies and professional identity play important roles in the socialization process of medical trainees and their level of professionalism; a form of social contract with the public to deliver quality care that all physicians commit to uphold (8). Therefore, the results of our study sheds light on the professional development of the physician workforce, which the public expects to see.

### Background

Miller’s pyramid, a broadly utilized framework for assessing medical trainees competence, describes four levels of knowledge and skills: Knows, Knows how, Shows how and Does (9). Through the lens of this pyramid, medical trainees’ competence and performance can be assessed from foundational medical knowledge (Knows) towards functioning independently in clinical situations (Does). Despite the framework receiving tremendous support in the educational setting as a very useful tool in facilitating teaching and learning (10), it has been criticized for its static nature which impedes it from reflecting performance in the real workplace, where dynamic factors are at play (4). Therefore, Cruess et al. (4) amended Miller’s pyramid includes the fifth level “Is” at its apex depicting professional identity formation. Thus, incorporating the process of developing values, attitudes and beliefs of healthcare professionals, together with the process of developing their competencies is of clinical importance. Consequently, this new Cruess et al. (4) framework proposes that the learning outcome in the workplace can represent a medical trainee’s professional development trajectory moving from “doing” to “being.”

#### Professional identity

According to Cruess et al. (4), the state of “being” and “feeling” like a physician are the hallmark of professional identity. Indeed, a growing body of research drawing from social constructionism, suggests that professional identities constitutes of attitudes, professional codes, beliefs and values that are characteristics of a profession, internalized by members through ongoing interactions and dynamic co-construction of their work experiences (11, 12). Likewise, building on this dynamic conceptualization of professional identities, Chang et al. (13) documented the complexity of emergency physicians’ work scope and work environment with its implications on the construction of their professional identities. Uncovering five key conceptual metaphors; safety net, gateway, market, sports and war that emergency physicians use to ascribe the value of and meaning to emergency care, the authors concluded that the metaphors give insights into the scaffolds that emergency physicians use to construct their multifaceted professional identities (13).

In a subsequent study which used a different sample, Chang et al. (14) teased out the dominating perspectives that emergency physicians held about their professional identities by analyzing the priorities they place on their previously reported roles, ethical codes, standards, values and beliefs. The results indicated four main viewpoints which underpin emergency physician’s professional identities. Viewpoint 1- *skills acquisition, capabilities and practical wisdom;* Viewpoint 2- *coping ability and resilience;* Viewpoint-3*: professional recognition and self-esteem; and* Viewpoint 4*: wellbeing and quality of life* (14). Viewpoint 1 highlighted having competence necessary to handle the challenges in emergency daily practice and the unpredictable working environment distinguished emergency specialties from other healthcare professions. Viewpoint 2 focused on career prospects and prioritized the importance personal physical strength and department leadership by which emergency physician can work under pressure and in charge of sudden events that arise at work. Viewpoint 3 accentuated being recognized within the institution and by the larger public can support the role emergency physicians play in the larger social context. Viewpoint 4 spotlighted to prioritize non-work commitments such as family and personal amusement and maintain the work-life balance can mitigate work pressure and contribute their success at work. Overall, this conceptualization of professional identity underpinned by context dependent co-construction and negotiation of values and beliefs lends itself to the operationalization of the professional identities of emergency physicians, a group of healthcare professions with multidisciplinary expertise whose work encompasses providing undifferentiated care to patients with acute care needs. It also echoes with Cruess’s conceptualization of the complex relationship between doing” and “being” a physician shaped by the interplay between the learning environment, individual identities, social interactivities and experiences.

#### Competency-based assessments

CBME as a successful innovation continues to be a key strategy in educating future doctors and other healthcare professionals (15, 16). Conducting valid and robust assessments in graduate medical education is essential for effective feedback and prepares the learner’s readiness for practice in real and complex clinical settings (2, 17). Thus, well-grounded assessment is a social responsibility among healthcare professionals to ensure quality of care is provided by the medical community. To date, such assessments include milestone assessments and entrustable professional activities.

The Accreditation Council for Graduate Medical Education (ACGME) milestones guidebook has highlighted that residency training programs need to emphasize the top level of the Miller’s pyramid “Does” which refers to work-based assessments (18). Milestones are educational outcomes and observable performance markers for residents professional development (19). Six core-competency of milestone assessment namely patient care and procedural skills, medical knowledge and clinical reasoning, professionalism, interpersonal and communication skills, practice-based learning and improvement, and systems-based practice have been recommended by the ACGME (18). Evidence shows the Miller’s Pyramid assessment outcome and the ACGME six core-competencies are significantly related (10).

Likewise, entrustable professional activities (EPAs) which are independent executable key tasks residents need to perform in their respective medical specialties, are fundamental to a profession and entrusted to a trainee to complete without supervision (20, 21). Entrustment decision implies clinical educators accept the inherent risks related to the clinical task and their judgement of the level of supervision needed for medical trainees to deliver good quality of care. Thus, EPAs are beyond the observation of the “Does” level of practice in Miller’s pyramid.

For residents, milestones are educational outcomes and observable performance markers in their professional development (19), while EPAs are independent executable key tasks residents need to perform in their respective medical specialties. The integrated frameworks of milestones and EPAs are expected to define and determine progression trajectory of individuals’ clinical competencies in the workplace (20, 22). Through the strategy of mapping, evidence has shown milestones are closely linked to EPAs by creating a unified structure that prepares residents to complete specified tasks effectively and autonomously in clinical practice (20, 22–24). However, despite the availability of such method, empirically there still exists the gap to confirm the link between milestone competency and EPA assessment across various medical specialties. Previous studies indicate a potential association between the two assessments (25, 26). Based on the empirical evidence, we hypothesize that milestone assessment is related to EPA assessment outcomes. Knowing such relationship in this study provides supervisors with a shared mental model of performance expectations for trainees (27, 28), reduces undesirable variability and improves assessment accuracy by giving specific feedback with regards to a resident’s professional development (29).

#### Professional identity and competency-based assessments

Using the Miller’s pyramid (9), and Cruess’s amended pyramid as a guide in this study (4), we argue that continual understanding of the learning outcomes’ progression, and the relationships between competency-based assessment and professional identity measure is paramount. Nevertheless, few studies have investigated the relationship of competency-based assessment on professional identities among healthcare professions (30, 31). To add to the literature, our study examined how emergency medical residents (EMRs) self-assess their clinical performance for quality of patient care by using milestone-based competencies and EPA assessment tools, and how these assessments relate to how EMRs perceive themselves as emergency physicians.

Therefore, we hypothesized that milestone-based core competencies and EPA assessment tools will positively correlate with professional identity. The understanding of the association provides evaluative judgement of task-specific clinical skills and the professional identity residents possess to stakeholders of CBME medical residency training programs.

## Methods

### Design and setting

A cross-sectional study of EMRs was conducted between April and July 2021 at 12 tertiary teaching hospitals across Taiwan with Residency Review Committee (RRC) approved emergency medicine residency programs. This study is part of a multi-year research regarding the incorporation of CBME and its subsequent assessment methods in emergency medicine residency training programs in Taiwan. Recently, Taiwan has been recognized as an international hub for ACGME regional faculty development (32).

### Participants

EMRs were invited through emails and posters pasted in the emergency department offices. Program directors of 46 RRC approved emergency residency training programs were further contacted to connect the research team with interested participants. Out of the 46 RRC approved programs, only those that had an ongoing implemented milestone and EPA assessment protocol were included as the final sample recruitment. EMRs of all residency years were recruited using a convenience sampling. Written informed consents were obtained from participants. The consent forms were collected directly if participants had the time to complete the questionnaire at the study site. If not, they were mailed back to the investigators. Participation was voluntary and anonymity was guaranteed through the use of identification numbers. The study was approved by the Chang Gung Medical Foundation Institutional Review Board (No: 201900082B0C601).

### Data collection and instruments

An online self-reported questionnaire using Google link was used for data collection and required approximately 20 min to complete. Considering this study was part of a multi-year project until July 2022, participants completed the questionnaire at their convenience. However, a follow-up was initiated to participants who delayed. The questionnaire included:

#### Milestones-based ACGME core competencies

The Chinese version of the Emergency Medicine Milestone (EMM) developed in 2014 by the Taiwan Society of Emergency Medicine (TSEM) using standardized consensus methods was used for assessment (33). In 2018, a revised version of this milestone assessment tool was introduced for the training of EMRs in Taiwan (34). EMM measures the stages that mark the development and acquisition of residents’ competencies using 23 sub-competency items which are aggregated into the ACGME six-core competencies. These include patient care (PC, 14 items), medical knowledge (MK, 1 item), professionalism (PROF, 2 items), interpersonal and communication skills (ICS, 2 items), practice-based learning and improvement (PBLI, 1 item), and system-based practice (SBP, 3 items) (34–36). A detailed list of the ACGME six-core competencies and their associated 23 sub-competencies are provided in supplementary file (Supplementary Table S1). The 23 sub-competency items are measured on a 5-level scale of proficiency with a higher level indicating higher competency (34, 35). Specifically, level 4 has been designed as the graduation target for residents (33).

#### Entrustable professional activities

EPAs were assessed using the Chinese version of the EPA scale developed by the TSEM (37). The Chinese EPA scale contains seven entrustable activities and each item is measured on a 5-level of proficiency with a higher level representing a higher competency (37). The seven items include: item 1—Management of out-of-hospital cardiac arrest patients; item 2—Management of shock patients; item 3—Management of patients with major trauma; item 4—Management of poisoned patients; item 5—Management of patients with acute chest pain; item 6—Management of patients with acute consciousness change; and item 7—Management of patients with acute dyspnea (37).

#### Professional identity

The Chinese version of the Emergency Physician Professional Identity and Value Scale (EPPIVS) was used to measure PI (Supplementary Table S2). The EPPIVS is a 20-item, self-reported questionnaire on a 7-point Likert scale. Underpinned by a construction of theoretical frameworks, the questionnaire was developed by a team of emergency physicians and other professionals in Taiwan (14). The 20 items measure four domains of professional identity: (1) skills acquisition, capabilities, and practical wisdom—7 items, (2) coping ability and resilience—4 items, (3) professional recognition and self-esteem—5 items, and (4) well-being and quality of life—4 items (14). With 150 participants, the initial scale reported a Cronbach’s α of ≥0.76 for all domains amongst emergency physicians (EPs) and EMRs in a single healthcare system. Evidence shows professional identity is multi-faceted or multi-domain and influenced by both internal and external factors (11, 14, 38, 39).

#### Demographic characteristics

The demographic characteristics measured included age, gender, marital status, highest degree attained, current residency year, hours worked/month, frequency of milestone and EPA evaluation within a 3-month training span, clinical, teaching and research workload, and number of residents in the program and in the same year of residency training.

### Data analysis

Analysis was performed using SPSS 24 (SPSS, Chicago, IL, United States). Descriptive statistics including frequency with percentage and mean with standard deviation (SD) were used to summarize participants’ characteristics after testing for normality. Bivariate analysis using chi-square test for categorical variables or a one-way analysis of variance (ANOVA) for continuous variables was performed to compare participants’ demographics and their residency years (40). Pearson correlation test was used to examine the relationship between the competency-based assessments**—**milestone and EPA, and professional identity. For better interpretation, the results of the 23 sub-competency milestone assessments were aggregated to form the ACGME six core competency results (18). Correlations (r) ranging 0.10 ~ 0.29 were categorized as small, 0.30 ~ 0.49 as medium and 0.50 ~ 1.00 as large (41). A value of p of ≤0.05 was considered as significant.

## Results

### Participant characteristics

The mean age of participants was 30.8 ± 2.7 years (Table 1). Of the 171 EMRs, 131 (76.6%) were male, 127 (74.3%) single, 164 (95.9%) had a bachelor’s degree and 115 (67.3%) worked >180 h/month. The participants were equally distributed across their residency years with the average of 4.4 ± 2.6 belonging to the same class. Overall, the average number of residents in-training was 16.9 ± 9.7. The mean reported percentage of workload for clinical, teaching and research was 82.0 ± 14.3, 12.7 ± 8.8, and 5.0 ± 4.8, respectively. Likewise, the mean number of milestone and EPA assessments conducted among the participants within the past 3 months were 9.3 ± 16.6 and 7.9 ± 12.5, respectively. The chi-square test or ANOVA showed no statistically significant differences (*p* = 0.10 ~ 0.99), except for age when comparing participants’ characteristics with their residency years of practice.

**Table 1:** Participant demographics across residency years (*N* = 171).

	Overall		Year 1		Year 2		Year 3		Year 4		*F/Χ^2^*	*p*	mean	(SD)	mean	(SD)	mean	(SD)	mean	(SD)	mean	(SD)		
Age (years)	30.8	(2.7)	29.6	(2.5)	29.9	(1.6)	30.7	(1.1)	32.9	(3.4)	18.3	<0.001
EPA evaluation (<3 months)	7.9	(12.5)	7.7	(11.1)	8.5	(13.3)	9.7	(15.7)	5.7	(9.3)	0.76	0.52
Milestone evaluation (<3 months)	9.3	(16.6)	7.8	(12.9)	13.8	(24.7)	8.8	(13.6)	6.6	(11.6)	1.58	0.2
Proportion of workload												
Clinical	82.0	(14.3)	79.0	(20.2)	86.4	(9.5)	81.5	(11.8)	81	(12.6)	2.14	0.1
Teaching	12.7	(8.8)	13.1	(8.5)	10.2	(7.3)	13.4	(9.1)	14.1	(10.0)	1.65	0.18
Research	5.0	(4.8)	3.9	(4.4)	4.4	(4.2)	6.1	(4.9)	5.5	(5.5)	2.00	0.12
Number of current residents	16.9	(9.7)	17.1	(9.8)	16.9	(10.5)	16.7	(8.4)	17.0	(10.4)	0.02	0.99
Residents in the same class	4.4	(2.6)	4.5	(2.4)	4.6	(2.9)	4.9	(2.3)	3.7	(2.6)	1.78	0.15
Gender												
Male (%)	131	(76.6)	33	(76.7)	33	(76.7)	34	(81.0)	31	(72.1)	0.93	0.82
Female (%)	40	(23.4)	10	(23.3)	10	(23.3)	8	(19.0)	12	(27.9)		
Marital status												
Single (%)	127	(74.3)	36	(83.7)	35	(81.4)	29	(69.0)	27	(62.8)	6.72	0.08
Married (%)	44	(25.7)	7	(16.3)	8	(18.6)	13	(31.0)	16	(37.2)		
Degree												
Bachelors (%)	164	(95.9)	42	(97.7)	40	(93.0)	42	(100.0)	40	(93.0)	3.96	0.27
Masters (%)	7	(4.1)	1	(2.3)	3	(7.0)	0	(0.0)	3	(7.0)		
Work hrs./month												
≤180 (%)	56	(32.7)	13	(30.2)	13	(30.2)	11	(26.2)	19	(44.2)	3.62	0.31
>180 (%)	115	(67.3)	30	(69.8)	30	(69.8)	31	(73.8)	24	(55.8)		

SD, Standard deviation; EPA, Entrustable Professional Activities.

### Correlation of competency-based assessments

**Figure 1:**
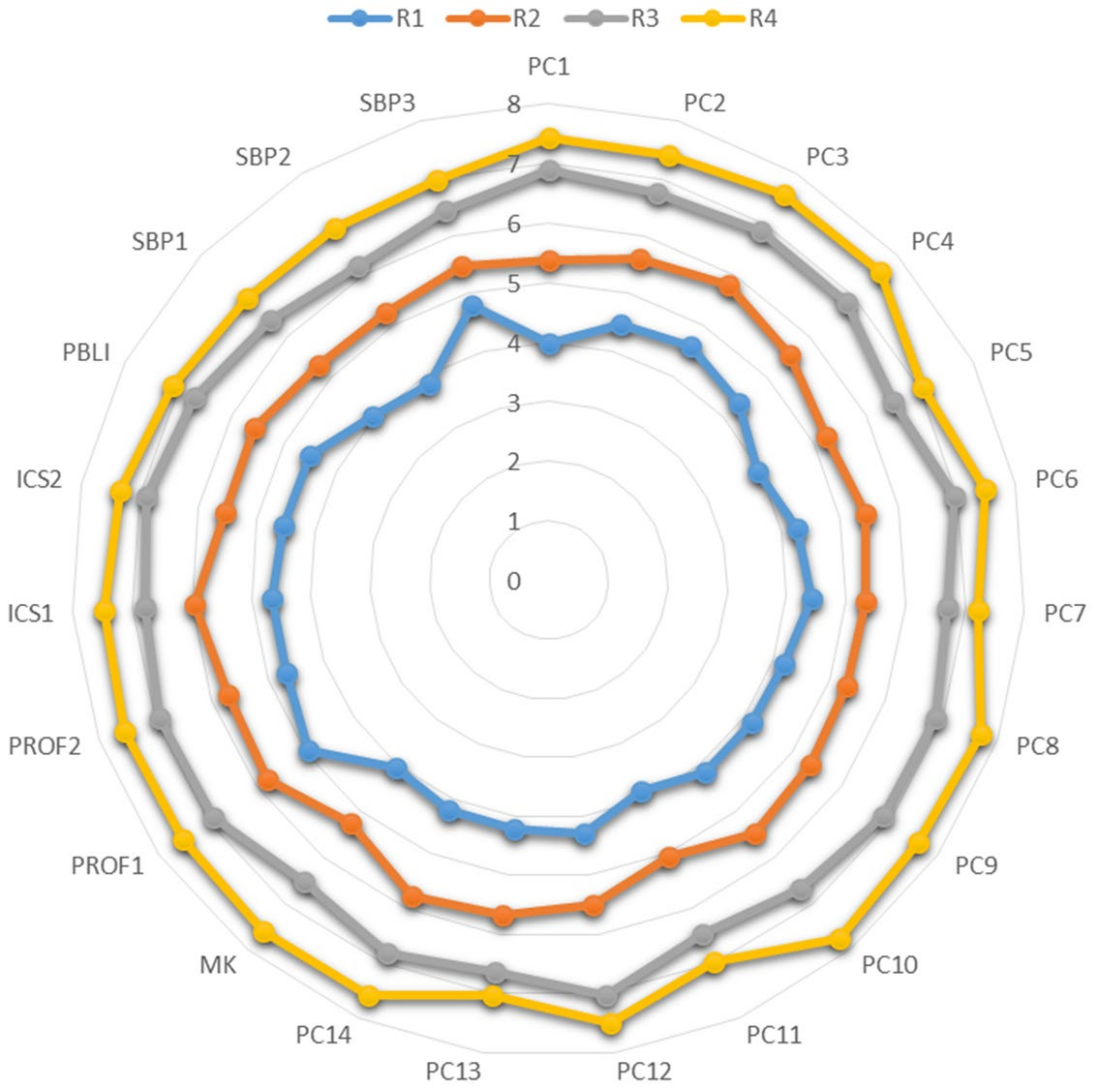
Radar plots of residency years with milestone-based competency assessment.

**Figure 2:**
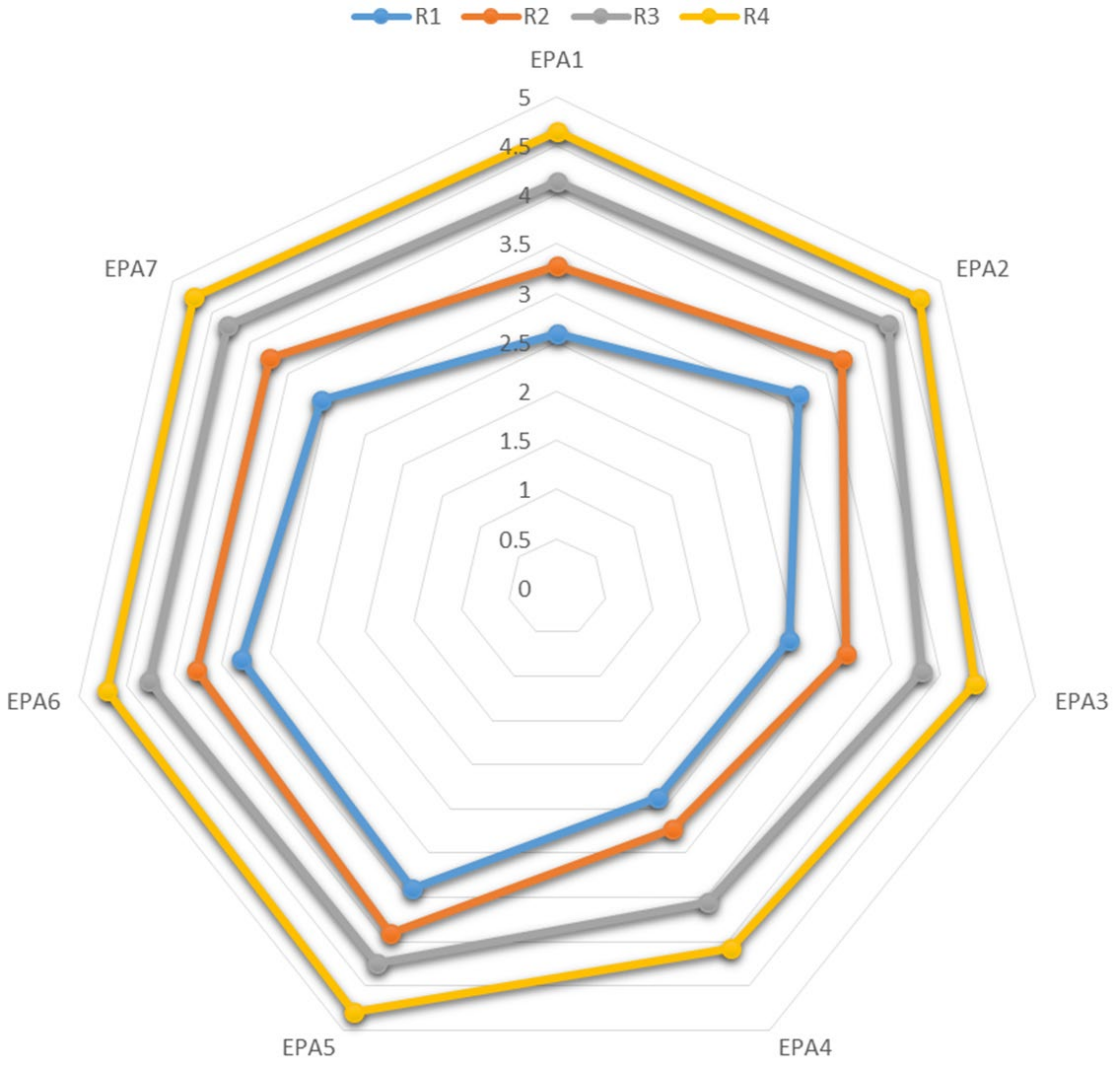
Radar plots of residency years with EPA assessment.

To assess the relationship between the ACGME milestone-based core competencies and EPA, a correlation analysis was performed. A significant positive correlation was demonstrated between the ACGME six milestone-based core competencies and the 7 EPA items (*r* = 0.40 ~ 0.74, *p* < 0.01) (Table 2). In Figures 1, 2, the radar plots illustrate the profiles of self-assessed milestones and EPAs with residency years. The results show residents self-assessment of milestone and EPA scores significantly increased with residency years.

**Table 2:** Correlation of milestone-based competencies and EPA assessment.

	PC	MK	PROF	ICS	PBLI	SBP
EPA1	0.74^**^	0.68^**^	0.63^**^	0.63^**^	0.53^**^	0.62^**^
EPA2	0.69^**^	0.64^**^	0.58^**^	0.58^**^	0.49^**^	0.53^**^
EPA3	0.73^**^	0.69^**^	0.62^**^	0.63^**^	0.59^**^	0.53^**^
EPA4	0.69^**^	0.64^**^	0.62^**^	0.60^**^	0.57^**^	0.60^**^
EPA5	0.62^**^	0.57^**^	0.53^**^	0.49^**^	0.40^**^	0.47^**^
EPA6	0.65^**^	0.56^**^	0.57^**^	0.54^**^	0.42^**^	0.51^**^
EPA7	0.70^**^	0.62^**^	0.61^**^	0.59^**^	0.50^**^	0.55^**^

^**^p < 0.01; ^*^p ≤ 0.05; PC, Patient care; MK, Medical Knowledge; PROF, Professionalism; ICS, Interpersonal and communication skills; PBLI, Practice-based learning and improvement; SBP, System-based practice; EPA, Entrustable professional activities.

### Correlation of professional identity and competency-based assessments

The professional identity questionnaire used in this study reported a Cronbach’s α of ≥0.70 for the subscales. However, the scores of the four dimensions of professional identities remained constant against residency years. The correlation between professional identity subscales with milestone-based core competencies and EPA showed a positive relationship (Table 3). The skills acquisition, capabilities, and practical wisdom domain of professional identities was positively correlated with 4 ACGME milestone-based core competencies including patient care, medical knowledge, practice-based learning and improvement, and system-based practice (*r* = 0.18 ~ 0.21, *p* ≤ 0.05) and 6 items of EPA (*r* = 0.16 ~ 0.22, *p* < 0.05). Additionally, the professional recognition and self-esteem domain of professional identities was positively correlated with 2 ACGME milestone-based core competencies including practice-based learning and improvement and system-based practice (*r* = 0.16 ~ 0.19, *p* < 0.05).

**Table 3:** Correlation of professional identity with milestone-based competencies and EPA assessment.

	SACPW	CAR	PRSE	WBQoL
PC	0.18^*^	−0.01	0.08	0.04
MK	0.21^*^	0.04	0.13	0.09
PROF	0.14	0.01	0.13	0.12
ICS	0.14	0.05	0.15	0.11
PBLI	0.20^*^	0.06	0.19^*^	0.14
SBP	0.18^*^	0.02	0.16^*^	0.08
EPA1	0.17^*^	−0.04	0.05	−0.01
EPA2	0.22^*^	−0.00	0.03	0.00
EPA3	0.16^*^	−0.06	0.03	0.04
EPA4	0.21^*^	0.02	0.08	0.05
EPA5	0.16^*^	0.01	0.01	−0.04
EPA6	0.15	−0.01	0.05	0.03
EPA7	0.17^*^	−0.05	0.06	−0.00

^*^p ≤ 0.05; SACPW, Skills acquisition, capabilities, and practical wisdom; CAR, Coping ability and resilience; PRSE, Professional recognition and self-esteem; WBQoL, Well-being and quality of life; PC, Patient care; MK, Medical Knowledge; PROF, Professionalism; ICS, Interpersonal and communication skills; PBLI, Practice-based learning and improvement; SBP, System based practice; EPA, Entrustable professional activities.

## Discussion

This multi-institutional survey is the first study to explore on EMRs self-evaluative judgement of their clinical skills trajectory using the ACGME milestone-based core competency and EPA assessment, and further, examine the association between task-specific clinical skills and professional identity. Using the amended Miller’s pyramid competency framework suggested by Cruess et al. (4), our study outcome advances the understanding of how EMRs perceive their readiness for practice by assessing the quality of “doing” and “being” an emergency physician in the emergency workplace, a unique clinical context under the social contract between emergency medicine and the public. Based on EMRs self-evaluation of their clinical skills, a significant positive relationship was observed between the ACGME six milestone-based core competencies and EPA assessment. Furthermore, the EMRs competencies indicate progression during the residency training years. However, their multi-faceted professional identities remained static. Only one dimension of professional identity**—**skills acquisition, capabilities, and practical wisdom, was weakly correlated to most milestone and EPA subscales or items. Our study, therefore, is an innovative implementation research bringing more understanding in regards to the intercorrelations between the ACGME milestone core competencies, EPA and professional identity to the stakeholders of CBME residency training programs.

The goal of medical education is to build a healthcare workforce that is able to provide high-quality care without requiring active supervision (29). Milestone alone provides more timely and specific assessment of healthcare provider performance and may enhance existing biannual competency-based formative feedback tools (25). Furthermore, connecting ACGME milestone reports to EPA-based evaluations allow training programs to use the competency assessment tools most related to their clinical context and assessors’ construct (20, 42, 43), while still apprehending broader competency progression for decisions of promotion and reporting to accrediting bodies (19, 23, 24, 44). With limited studies conducted on the association of milestone-based competence and EPA assessment, our finding adds to the empirical reliability and validity of competency-based assessments. Additionally, it shows that milestone and EPA assessments can be synergistically used in the workplace when evaluating EMRs (25). As expected, there exists a relationship between milestone and EPA as they both define residents’ performance trajectories (22). This finding is similar to previous studies that found a moderate to high positive correlation between milestones and EPA assessment in pediatric, internal medicine and surgical residency training, which is innovative for emergency medicine (25, 26, 45) Through our survey, milestones and EPA assessment were, therefore, designed to define the tasks and standards that can lead to entrustability and autonomy of EMRs (20). This can further provide a foundation towards developing effective educational interventions that increases residents’ performance in the clinical practice.

Evaluative judgement can be defined as the ability to determine the quality of work that has been completed by an individual and others in a specific context (46, 47). It reflects on the long-term impacts of assessment on the professional development of trainees and thereby, contributing to residents’ construct of their professional identity (48). It is evident from literature that knowledge, attitude and clinical skills acquisition are intercorrelated (49), which are important aspects that can further reinforce residents’ professional identity (3, 14). In the present study, the skills acquisition, capabilities, and practical wisdom domain of professional identity was weakly but comprehensively correlated to most competency-based assessments, which highlighted EMRs capabilities to multi-task, diagnose a medical condition quickly and experience the fresh challenges that the emergency department brings every day. In turn, this helps to make sense of their learning on how to act and behave like an emergency physician from co-workers in the emergency setting (14). Additionally, the professional recognition and self-esteem domain of professional identity was correlated to two milestone-based core competency subscales**—**practice-based learning and improvement, and system-based practice, showing EMRs ability to utilize systematic efforts to improve quality, safety or value of healthcare services, and to conduct interprofessional care and practice in a complex healthcare system necessary for their professional identity formation (14). Thus, the development of skills and a resident’s ability to learn, competently perform tasks and make sound medical-related decisions for quality of patient care at a larger context and system is of importance for EMRs and partly influences their professional identity (49). Evident from the lack of correlation found in this study between competency-based assessments and professional identity, one possible reason is that emergency medicine professional identities are multifaceted, constructed and impacted by experiencing different levels of sociocultural identification (11). We therefore, suggest the need to further develop and validate EMRs competency-based assessment tools to capture all the important aspects that affect physicians’ professional identity development.

Given that one fundamental aspect of community of practice is that members should have mutual engagement in tasks and shared goals, milestone-based competency and EPA assessments have been designed and implemented under consensus. This can be used to identify activities and competencies unique to a particular profession or specialty such as the emergency department (50). By being an active participant, competently performing the specified competencies, and reflecting on their progress, trainees’ professional identities are expected to form and develop over time (51). However, EMRs professional identity domains in this study remained static contrary to the task-specific competencies, which evolved with residency years. Considering our study participants were all residents in training in various residency years who provided medical care under supervision in different emergency institutions, their professional identity is in infancy and still developing. This is consistent with Cruess’s amended model of competence which considers the professional identity as the last stage of competence (4). Hence, the EMRs professional identities will continue to progress and be clearer as the gain additional work experiences either as an attending physician or clinical educator. Through a social identity lens, the construction of residents’ identity is a two way process of maintaining existing identities and gaining new identities toward social identity compatibility, which takes time to be developed and tested (52). Our study further adds knowledge into the emergency medicine community of practice in that milestone-based competency and EPA assessments are not major determining factors of professional identity.

The practical implications of our study come from the establishment of the relationship between milestone and EPA assessment in the clinical setting which facilitates effective clinical supervision for residents in various specialties. We suggest that milestones can be used in conjunction with EPA assessment. Exploring the association informs attending physicians or clinical educators to complement milestone and EPAs during assessment for a holistic evaluation. Instead of using the assessment tools separately, simultaneous use achieves a comprehensive and rigorous assessment. Likewise, using the competency assessment tools together allows residents to be informed of their competency trajectory in a reliable and comprehensive manner. This can increase their ability to make sense of their professional development trajectory; promoting their receptivity to feedback.

### Strengths and limitation

The strengths of our study lie in the recruitment of participants from various teaching hospitals in Taiwan that have RRC qualified emergency medicine residency training programs, which adds to the generalizability of the results. We also utilized the self-reported assessments following the scaffold recommended by the ACGME, which plays an important role for EMRs self-evaluative judgement to define their quality of work and future professional development. Likewise, through the lens of extending the Miller’s pyramid, we add to the gap and shed light on how to prepare medical training programs to concurrently promote clinical competence and professional identity development in the clinical workplace. However, our study is not without limitations, and careful considerations should be made when interpreting and applying the findings to other contexts. Possibly only 12 out of 46 RRC qualified training programs have fully implemented their CBME at a larger scale were enrolled in this study. However, this was a multi-institutional survey which recruited 171 EMRs across Taiwan and accounted for 40% of total training capacity in the 46 residency programs. Our study results therefore, can still be generalized to the residency population. We used a cross-sectional study to explore the relationship between milestone-based competency and EPA assessment, and professional identity, which was unable to show the cause-effect relationships. Future studies to consider longitudinal study designs and examine such cause-effect relationships by collecting and analyzing the objective workplace assessment data is needed. Despite these limitations, our study is the first to assess and provide a basis for understanding the link between competency-based assessment tools and professional identity among EMRs from an East Asian perspective.

## Conclusion

This study highlights the intercorrelation between milestone-based competency and EPA assessments with multifaceted professional identities among EMRs. The implementation of assessment methods for evaluating progress of medical trainees and professional identity is needed to assess their readiness for practice. A highly positive correlation existed between the competency-based assessment tools**—**milestone and EPA**—**indicating they can be synergistically used by supervisors and clinical educators to evaluate clinical performance during residency training. Despite theoretical evidence showing competence and professional identity go hand in hand, our study found that at the residency stage the association between task-specific competencies and professional identities is partial. This suggests that in addition to focusing on the acquisition of skills, EMRs still need strong support from their communities of practice to facilitate and reinforce the development of their professional identities during training. Therefore, further research is needed to understand how resident’s competency trajectory aligns with how they internalize different values that underpin their professional identities during clinical training.

## Data availability statement

The original contributions presented in the study are included in the article/Supplementary material, further inquiries can be directed to the corresponding author.

## Ethics statement

Ethical approval was obtained from the Chang Gung Medical Foundation Institutional Review Board (No: 201900082B0C601). Participants provided their written informed consent before participation.

## Author contributions

Y-CC and NN: conceptualization. Y-CC, NN, MK, and RC: methodology. MK, Y-CC, and C-HC: formal analysis and investigation. MK: writing—original draft preparation. Y-CC, MK, RC, and C-HC: writing—review and editing. Y-CC: supervision. All authors contributed to the article and approved the submitted version.

## Funding

This study was funded by Ministry of Science and Technology, Taiwan (Grant no. 108-2511-H-182-007-MY2).

## Conflict of interest

The authors declare that the research was conducted in the absence of any commercial or financial relationships that could be construed as a potential conflict of interest.

## Publisher’s note

All claims expressed in this article are solely those of the authors and do not necessarily represent those of their affiliated organizations, or those of the publisher, the editors and the reviewers. Any product that may be evaluated in this article, or claim that may be made by its manufacturer, is not guaranteed or endorsed by the publisher.
