# Supplementary material for: Emergency residents' self-perceived readiness for practice: the association of milestones, entrustable professional activities, and professional identities—a multi-institutional survey
Source: Front Med (Lausanne). 2023 May 12;10:1032516. doi: 10.3389/fmed.2023.1032516 (PMC10213224; doi:10.3389/fmed.2023.1032516)
Supplement: Supplementary file 1 [file Table_1.DOCX]

**List of supplementary files**

**Table S1.** Description of the ACGME core competencies and its associated milestone sub-competencies

| Core competencies | Milestone sub-competencies |
| --- | --- |
| 1. Patient care (PC) | PC1: Emergency Stabilization |
|  | PC2: Performance of Focused History & Physical Exam |
|  | PC3: Diagnostic Studies |
|  | PC4: Diagnosis |
|  | PC5: Pharmacotherapy |
|  | PC6: Observation and Reassessment |
|  | PC7: Transitions of Care |
|  | PC8: Task-switching |
|  | PC9: General Approach to Procedures |
|  | PC10: Airway Management |
|  | PC11: Anesthesia and Acute Pain Management |
|  | PC12: Emergency Ultrasound |
|  | PC13: Other Diagnostic & Therapeutic Procedures—Wounds Management |
|  | PC14: Other Diagnostic & Therapeutic Procedures—Vascular Access |
| 1. Medical Knowledge (MK) | Medical Knowledge |
| 1. Professionalism (PROF) | PROF1: Professional Values |
|  | PROF2: Accountability |
| 1. Interpersonal & Communication Skills (ICS) | ICS1: Patient Centered Communication |
|  | ICS2: Team Management |
| 1. Practice‐based learning and improvement (PBLI) | Practice Based Performance Improvement |
| 1. System‐based Practice (SBP) | SBP1: Patient Safety |
|  | SBP2: System-based Management |
|  | SBP3: Technology |

**Table S2.** Validated 20-item Emergency Physicians’ Professional Identities Value Scale (EPPIVS)

|  | Not at all the same |  |  |  |  |  | Pretty much the same |
| --- | --- | --- | --- | --- | --- | --- | --- |
| 1. Swift decision making around patients’  discharge | 1 | 2 | 3 | 4 | 5 | 6 | 7 |
| 2. Utilizing clinical skills that physicians in  other specialties are unfamiliar with | 1 | 2 | 3 | 4 | 5 | 6 | 7 |
| 3. Having the ability to trust colleagues | 1 | 2 | 3 | 4 | 5 | 6 | 7 |
| 4. Playing an important role in society | 1 | 2 | 3 | 4 | 5 | 6 | 7 |
| 5. Happiness in one’s personal life | 1 | 2 | 3 | 4 | 5 | 6 | 7 |
| 6. Utilizing personal values and beliefs to  sustain professional work | 1 | 2 | 3 | 4 | 5 | 6 | 7 |
| 7. Contributing as a good leader in  professional teams | 1 | 2 | 3 | 4 | 5 | 6 | 7 |
| 8. Gaining pleasure from professional work | 1 | 2 | 3 | 4 | 5 | 6 | 7 |
| 9. Maintaining standard of care, despite medical disputes | 1 | 2 | 3 | 4 | 5 | 6 | 7 |
| 10. Having a high level of emotional  intelligence for workplace effectiveness | 1 | 2 | 3 | 4 | 5 | 6 | 7 |
| 11. Development of professional subspecialties | 1 | 2 | 3 | 4 | 5 | 6 | 7 |
| 12. Rapid recovery from upset at work | 1 | 2 | 3 | 4 | 5 | 6 | 7 |
| 13. Remaining calm when managing sudden events at work | 1 | 2 | 3 | 4 | 5 | 6 | 7 |
| 14. Having a manageable workload | 1 | 2 | 3 | 4 | 5 | 6 | 7 |
| 15. Staying positive when facing patients’  complaints | 1 | 2 | 3 | 4 | 5 | 6 | 7 |
| 16. Efficient multitasking | 1 | 2 | 3 | 4 | 5 | 6 | 7 |
| 17. Staying positive when working under  pressure | 1 | 2 | 3 | 4 | 5 | 6 | 7 |
| 18. Managing a wide range of medical  conditions | 1 | 2 | 3 | 4 | 5 | 6 | 7 |
| 19. Diagnosing medical conditions quickly | 1 | 2 | 3 | 4 | 5 | 6 | 7 |
| 20. Managing challenging patients effectively | 1 | 2 | 3 | 4 | 5 | 6 | 7 |

Subscale 1: Skills Acquisition, Capabilities and Practical Wisdom (Items 1, 2, 9, 16, 18, 19, 20); Subscale 2: Coping Ability and Resilience (Items 3, 12, 13, 17); Subscale 3: Professional Recognition and Self –Esteem (Items 4, 7, 8, 11, 15); Subscale 4: Well 4: Well -Being and Quality of Life (Items 5, 6, 10, 14).
